# Supplementary material for: AKAP5 complex facilitates purinergic modulation of vascular L-type Ca2+ channel CaV1.2
Source: Nat Commun. 2020 Oct 20;11:5303. doi: 10.1038/s41467-020-18947-y (PMC7575592; doi:10.1038/s41467-020-18947-y)
Supplement: Supplementary file 1 — Supplementary Information [file 41467_2020_18947_MOESM1_ESM.pdf]

Supplementary Information

AKAP5 complex facilitates purinergic modulation of vascular L-type Ca^{2+} channel $Ca_V1.2$

Maria Paz Prada[1*], Arsalan U. Syed[1*], Gopireddy R. Reddy[1*], Miguel Martín-Aragón Baudel[1], Víctor A. Flores-Tamez[1], Kent C. Sasse[2], Sean M. Ward[3], Padmini Sirish[4], Nipavan Chiamvimonvat[1, 4, 5], Peter Bartels[1], Eamonn J. Dickson[6], Johannes W. Hell[1], John D. Scott[7], Luis F. Santana[6], Yang K. Xiang[1, 5], Manuel F. Navedo[1], and Madeline Nieves-Cintrón[1]

[1]Department of Pharmacology, University of California Davis, Davis, CA, USA 95616, [2]Sasse Surgical Associates, Reno, NV, USA 89502, [3]Department of Physiology and Cell Biology, University of Nevada Reno, Reno, NV, USA 89557, [4]Department of Internal Medicine, University of California Davis, Davis, CA, USA 95616, [5]VA Northern California Healthcare System, Mather, CA, USA 95655, [6]Department of Physiology and Membrane Biology, University of California Davis, Davis, CA, USA 95616, and [7]Department of Pharmacology, University of Washington Seattle, Seattle, WA, USA 98195

Supplemental Figures and Tables

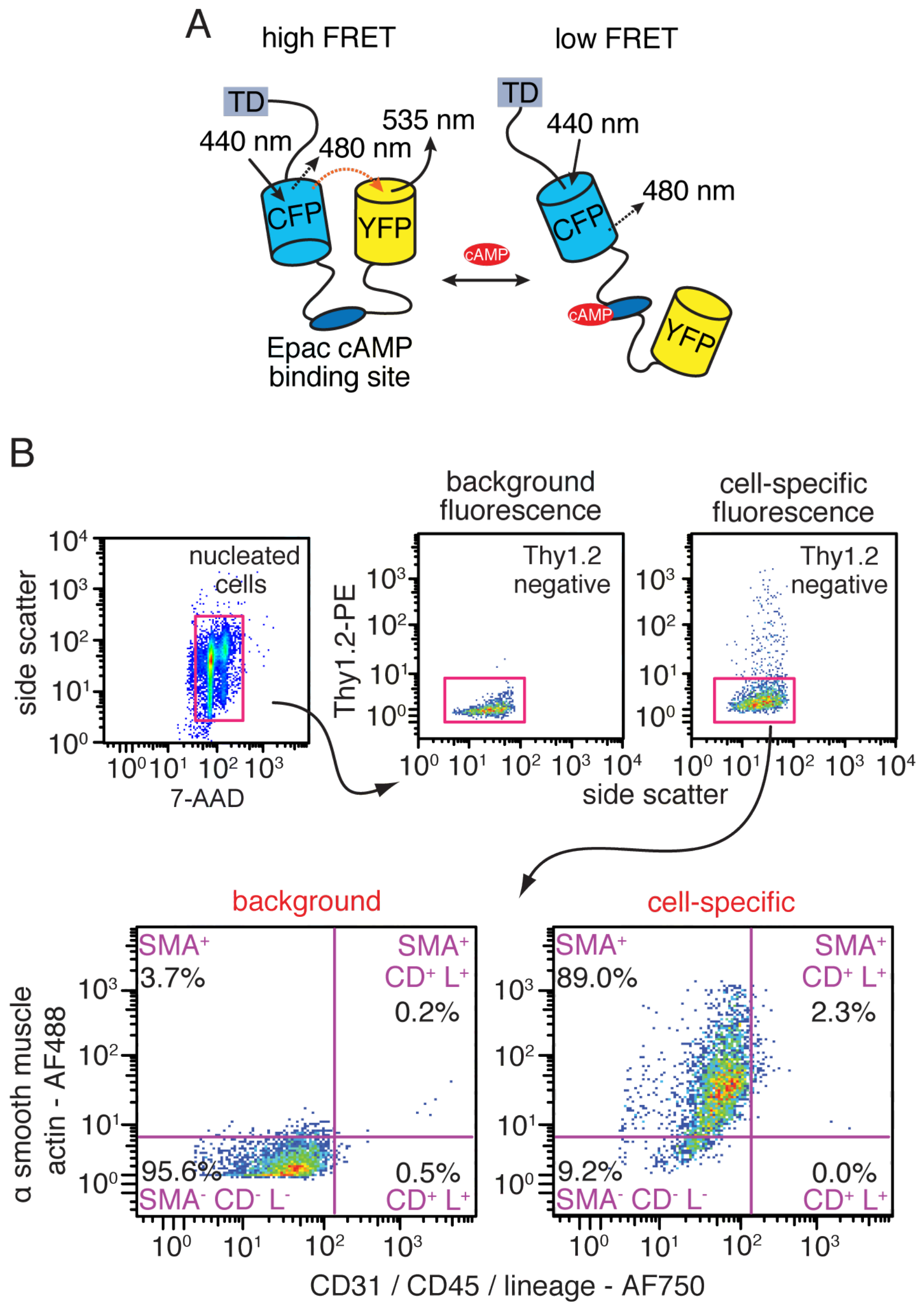

Supplementary Figure 1: Diagram of the ICUE3-PM sensor and flow cytometry analysis of unpassaged primary cultured arterial myocytes. A) Schematic of the design for the plasma membrane-targeted Epac1-camps-based FRET sensor (ICUE3-PM). TD = targeting domain. **B)** Flow cytometry analysis of arterial myocytes (α-

SMA$^+$/Thy$^-$/CD31$^-$/CD45$^-$/Lin$^-$). Nucleated cells were selected from the mixed population based on the incorporation of 7-AAD (top left panel). Selection of non-fibroblasts (Thy1.2^{neg}) are shown in the top middle and right panels. Arterial myocytes were identified as CD31$^-$/CD45$^-$/lineage negative and α-smooth muscle actin positive in the bottom panels. X and Y-axes represent arbitrary units. Representative results are shown (n = 4 preparations). Source data are provided as Source Data file.

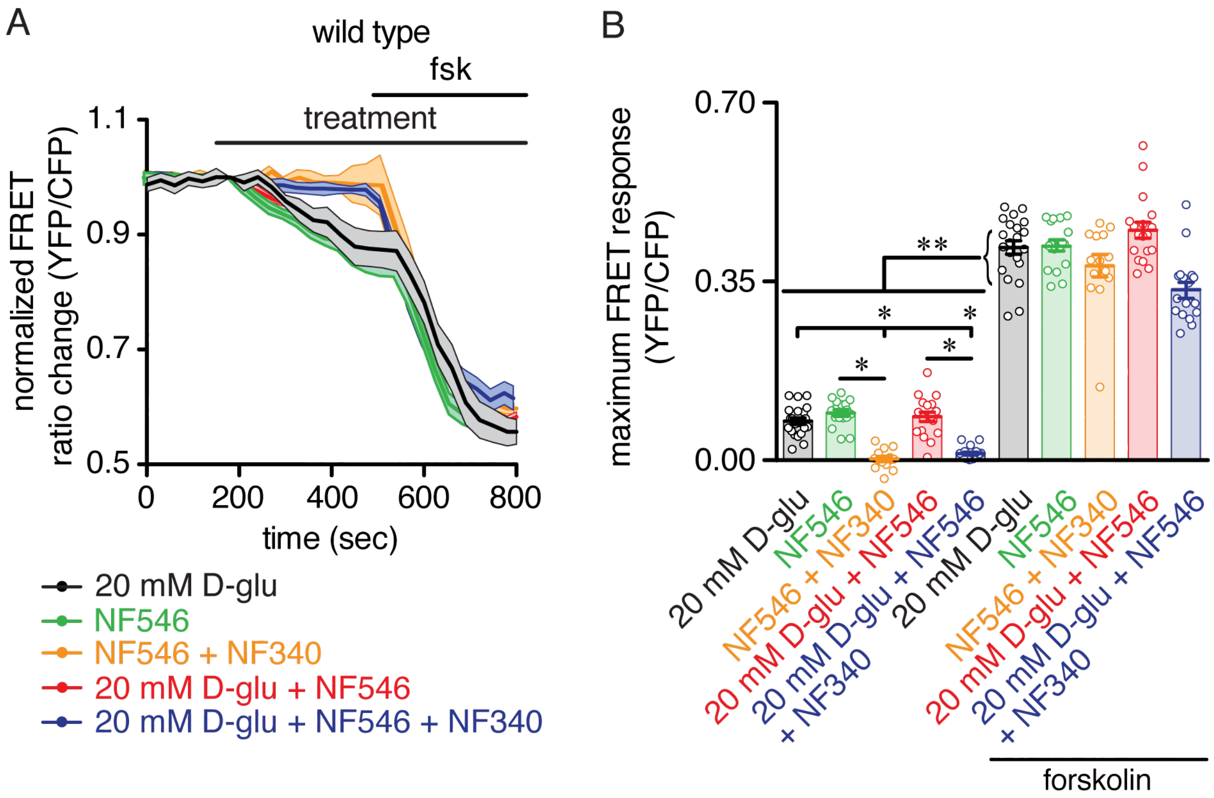

Supplementary Figure 2: NF340 prevents glucose/NF546-induced cAMP synthesis. A) Time courses of averaged ICUE3-PM traces (mean = solid line; SEM = shadow) in wild type (WT) arterial myocytes in response to 20 mM D-glucose (D-glu; black; n = 21 cells/6 mice), 500 nM NF546 (green; n = 17 cells/6 mice), and 20 mM D-glu + NF546 (red; n = 18 cells/6 mice) and after application of 1 μM forskolin. In a set of experiments, cells were first pretreated with the $P2Y_{11}$ antagonist NF340 (10 μM) before applying 500 nM NF546 (orange; n = 14 cells/6 mice) and 20 mM D-glucose + NF546 (blue; n = 17 cells/6 mice). Horizontal bar indicates treatment. **B)** Plot of maximum FRET responses (YFP/CFP) for arterial myocytes in response to the indicated treatment. *$P < 0.05$ with Kruskal-Wallis One-way ANOVA with Dunn's multiple comparisons. The single asterisks highlight significant differences between datasets in the absence of forskolin. The double asterisks indicate a statistical difference within the same experimental group in the absence and presence forskolin. $P = 0.0003$ for 20 mM D-glu-20 mM D-glu + NF546 + NF340, and all other significant P values are < 0.0001. Data represent mean ± SEM. Source data are provided as Source Data file.

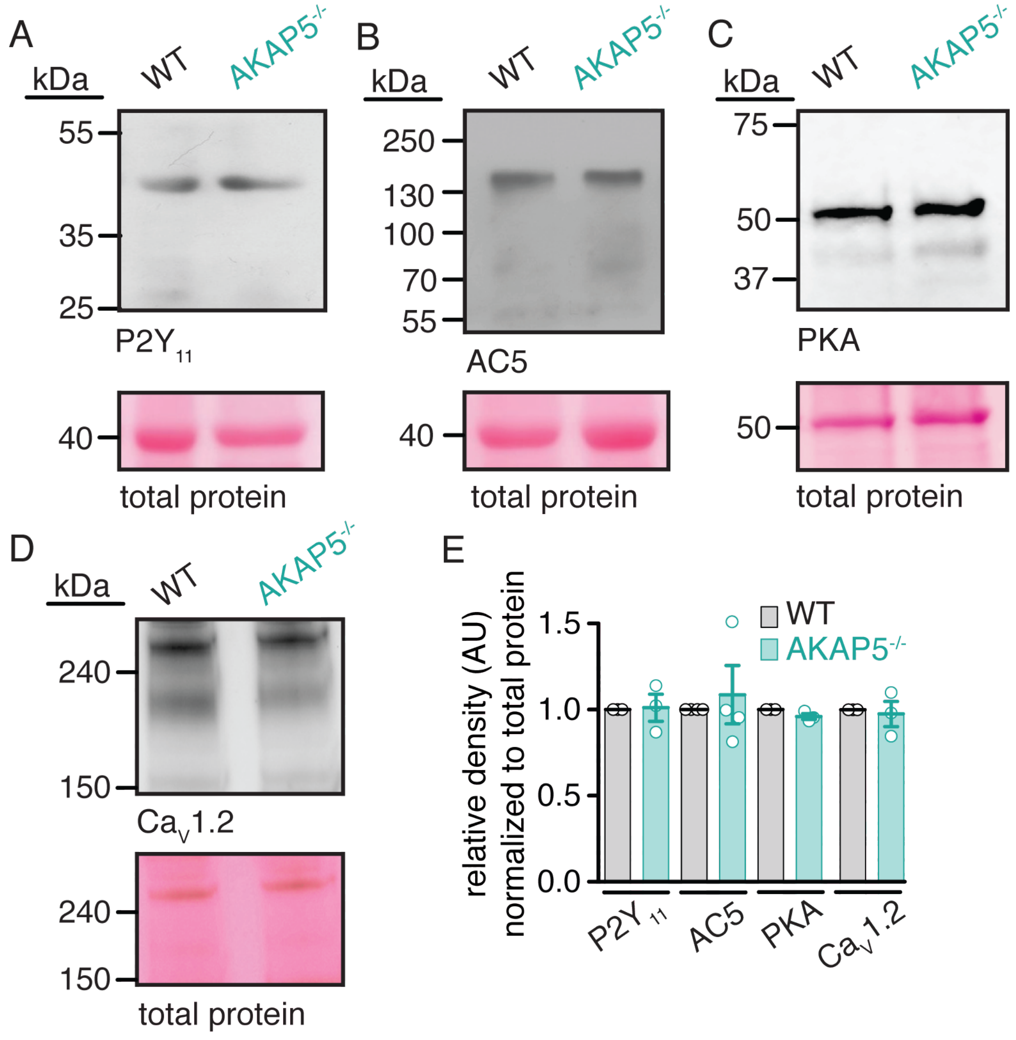

Supplementary Figure 3: Similar P2Y$_{11}$, AC5, PKA and Ca$_V$1.2 protein abundance in WT and AKAP5$^{-/-}$ lysates. Representative Western blot of immunoreactive bands of expected molecular mass for **A**) P2Y$_{11}$ (~40 kDa), **B**) AC5 (~130 kDa), **C**) PKA (~50 kDa) and **D**) Ca$_V$1.2 (~240 kDa) in wild type (WT) and AKAP5$^{-/-}$ lysates. Ponceau staining was used for normalization of samples to total protein. **E**) Amalgamated densitometry data for P2Y$_{11}$ (n = 3 lysates/3 mice), AC5 (n = 4 lysates/4 mice), PKA (n = 3 lysates/3 mice) and Ca$_V$1.2 (n = 3 lysates/3 mice) in WT and AKAP5$^{-/-}$ lysates. *P < 0.05 with two-tailed Mann-Whitney test comparing results between WT and AKAP5$^{-/-}$ samples. Data represent mean ± SEM. Source data are provided as Source Data file.

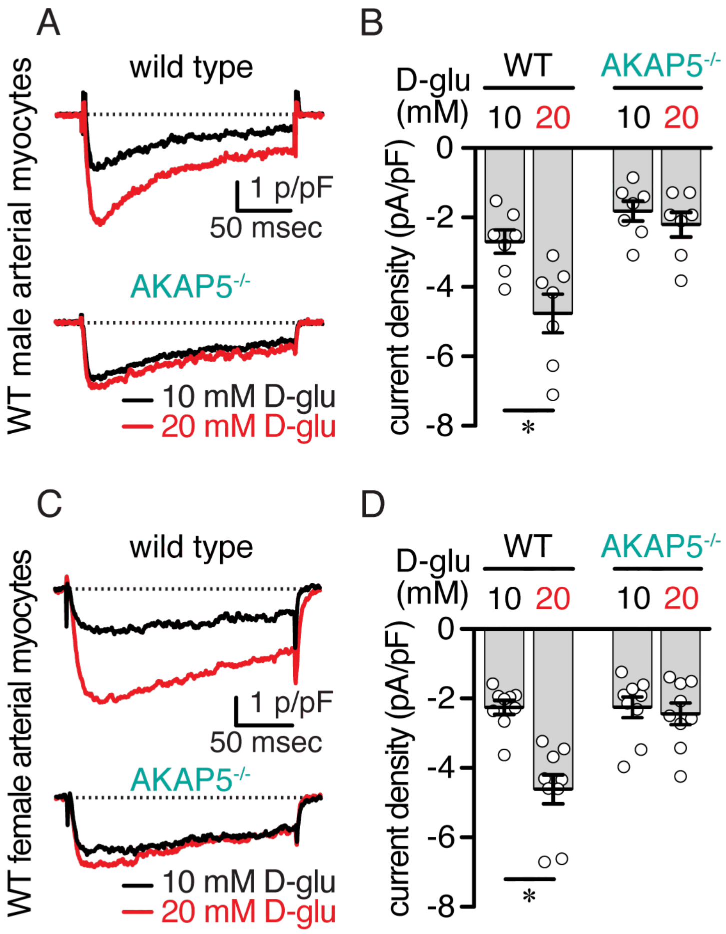

Supplementary Figure 4. AKAP5 is required for glucose-induced L-type Ca^{2+} channel potentiation in mouse male and female arterial myocytes. Representative Ba^{2+} currents (I_{Ba}) recordings and summary I_{Ba} data of cerebral arterial myocytes from wild type (WT) and AKAP5$^{-/-}$ **A, B)** male (n = 7 cells/5 WT mice and n = 7 cells/4 AKAP5$^{-/-}$ mice) and **C, D)** female (n = 9 cells/3 WT mice and n = 9 cells/3 AKAP5$^{-/-}$ mice) mice evoked by step depolarizations from -70 to +10 mV in the presence of 10 mM or 20 mM D-glucose (D-glu). Dotted lines reflect baseline. *$P < 0.05$ with two-tailed Wilcoxon test. Comparisons between the 10 mM and 20 mM D-glu condition for each dataset. $P = 0.0156$ for WT male 10 mM D-glu-20 mM D-glu and $P = 0.0039$ for WT female 10 mM D-glu-20 mM D-glu. Data represent mean ± SEM. Source data are provided as Source Data file.

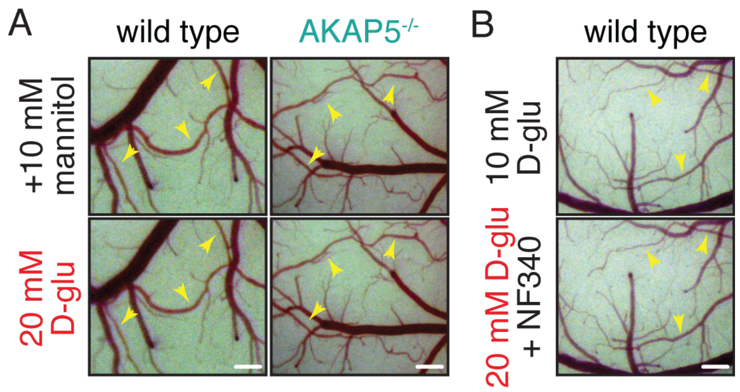

Supplementary Figure 5. Exemplary images of cerebral arteries visualized through a cranial window in WT and AKAP5$^{-/-}$ mice. Representative images of middle cerebral arteries and branches visualized through an open cranial window used to analyze diameter changes in response to **A)** 10 mM D-glucose (D-glu) + 10 mM mannitol and 20 mM D-glu in wild type (WT) and AKAP5$^{-/-}$ mice and **B)** 10 mM D-glu and 20 mM D-glu + NF340 in WT mice. Yellow arrows point to arteries. Scale bars = 50 pixels.

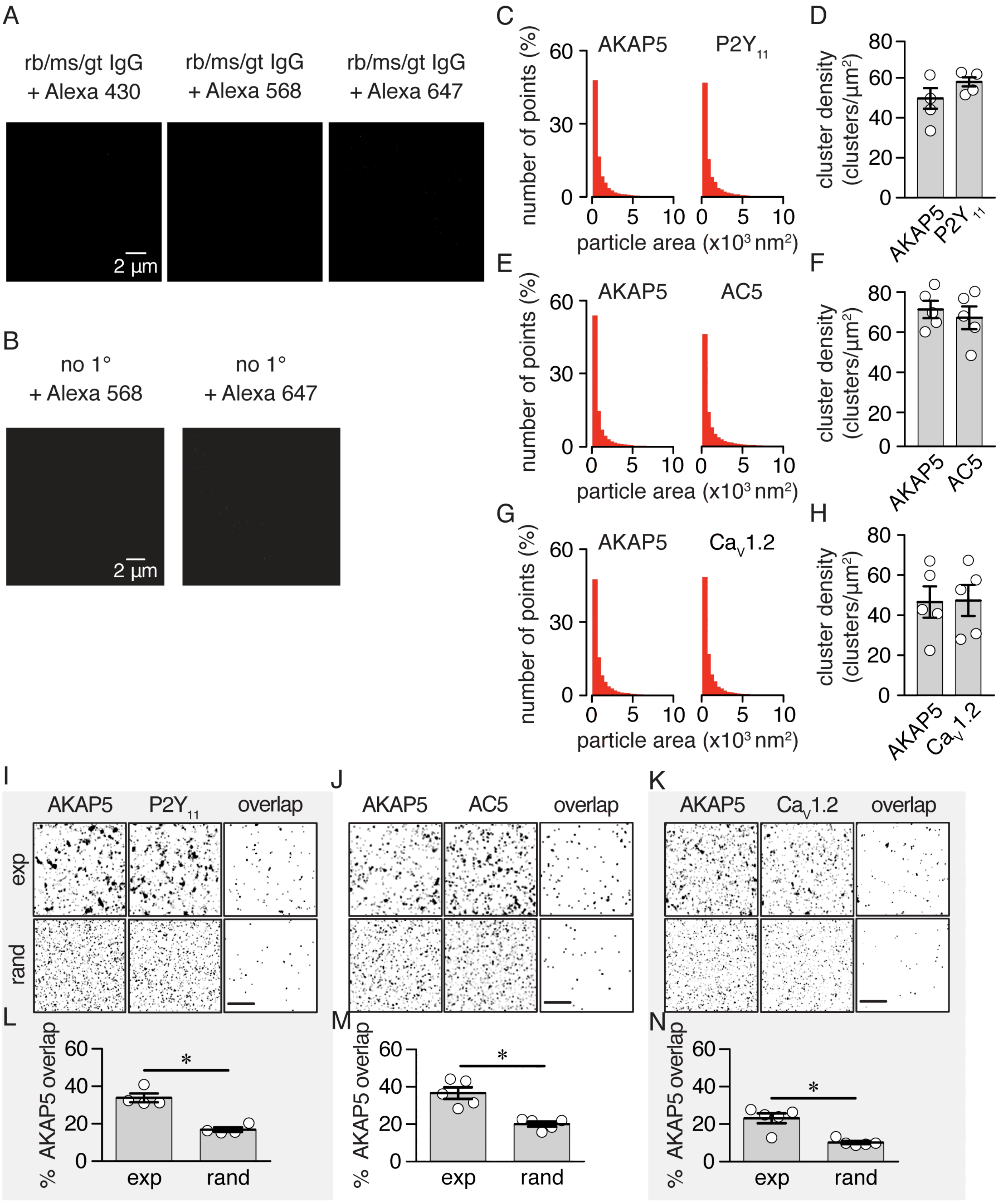

Supplementary Figure 6: Negative controls for Airyscan and GSD super-resolution imaging, particle area and cluster density analysis, and protein overlap analysis. A) Representative Airyscan images of an arterial myocyte triple labeled with

primary rabbit (rb), mouse (ms) and goat (gt) immunoglobulin G (IgGs) and secondary Alexa 430, Alexa 568 and Alexa 647 antibodies. Similar results were observed in 5 different cells from 2 mice. **B)** Exemplary Ground State Depletion (GSD) reconstruction maps of an arterial myocyte labeled only with secondary antibodies (no 1° + Alexa 568 and no 1° + Alexa 647). Similar results were observed in 7 different cells. Histograms of the cluster area and scatter plot of the cluster density of AKAP5 and $P2Y_{11}$ (**C, D**; n = 4 cells), AKAP5 and AC5 (**E, F**; n = 5 cells) and AKAP5 and $Ca_V1.2$ (**G, H**; n = 5 cells). Average cluster area for each dataset are **C)** AKAP5 = 2408 ± 46, $P2Y_{11}$ = 2697 ± 58, **E)** AKAP5 = 2721 ± 252, AC5 = 3514 ± 304 and **G)** AKAP5 = 2752 ± 52, $P2Y_{11}$ = 2093 ± 31. Representative experimental (exp; *top*) and randomized (rand; *bottom*) images of reconstruction maps for **I)** AKAP5-$P2Y_{11}$ (n = 4 cells), **J)** AKAP5-AC5 (n = 5 cells) and **K)** AKAP5-$Ca_V1.2$ (n = 5 cells). Scatter plots of percentage of overlap of AKAP5 with **L)** $P2Y_{11}$, **M)** AC5 and **N)** $Ca_V1.2$ for experimental and randomized simulation images. *P < 0.05 with two-tailed Mann-Whitney test. P = 0.0286 for AKAP5-$P2Y_{11}$, P = 0.0079 for AKAP5-AC5 and P = 0.0159 for AKAP5-$Ca_V1.2$. Data represent mean ± SEM. Source data are provided as Source Data file.

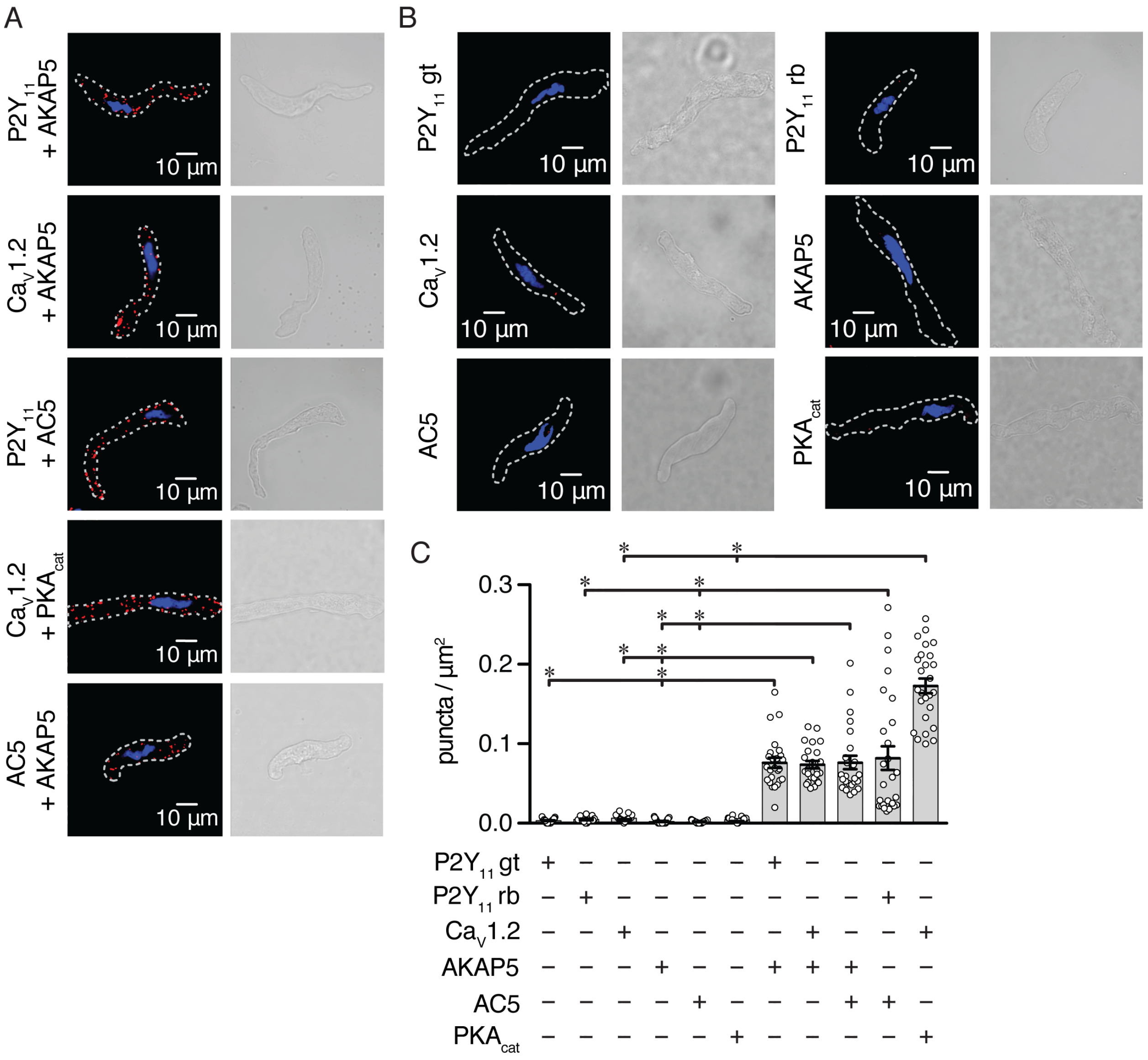

Supplementary Figure 7. An AKAP5/P2Y$_{11}$/AC5/PKA/Ca$_V$1.2 nanocomplex in human arterial myocytes. A) Representative merged fluorescence proximity ligation assay (PLA; red)/nucleus (blue; left panels) and differential interference contrast (right panels) images of human arterial myocytes co-labeled for **A)** P2Y$_{11}$ + AKAP5, Ca$_V$1.2 + AKAP5, P2Y$_{11}$ + AC5, Ca$_V$1.2 + PKA$_{cat}$ and AC5 + AKAP5, as well as representative images of cells labeled with only one 1° antibody for **B)** P2Y$_{11}$ (goat = gt), P2Y$_{11}$ (rabbit = rb), Ca$_V$1.2, AKAP5, AC5 and PKA$_{cat}$. **C)** Quantification of PLA fluorescent puncta per cell area (puncta/µm^2) for human arterial myocytes labeled for P2Y$_{11}$ gt (n = 27 cells/3 humans), P2Y$_{11}$ rb (n = 17 cells/3 humans), Ca$_V$1.2 (n = 19 cells/3 humans), AKAP5 (n

= 31 cells/3 humans), AC5 (n = 33 cells/3 humans), PKA_{cat} (n = 21 cells/3 humans), $P2Y_{11}$ + AKAP5 (n = 25 cells/3 humans), $Ca_V1.2$ + AKAP5 (n = 24 cells/4 humans), AC5 + AKAP5 (n = 25 cells/3 humans), $P2Y_{11}$ + AC5 (n = 27 cells/3 humans), and $Ca_V1.2$ + PKA_{cat} (n = 26 cells/3 humans). *$P < 0.05$ with Kruskal-Wallis One-way ANOVA with specific Dunn's multiple comparisons as displayed. All significant P values are < 0.0001. Data represent mean ± SEM. Source data are provided as Source Data file.

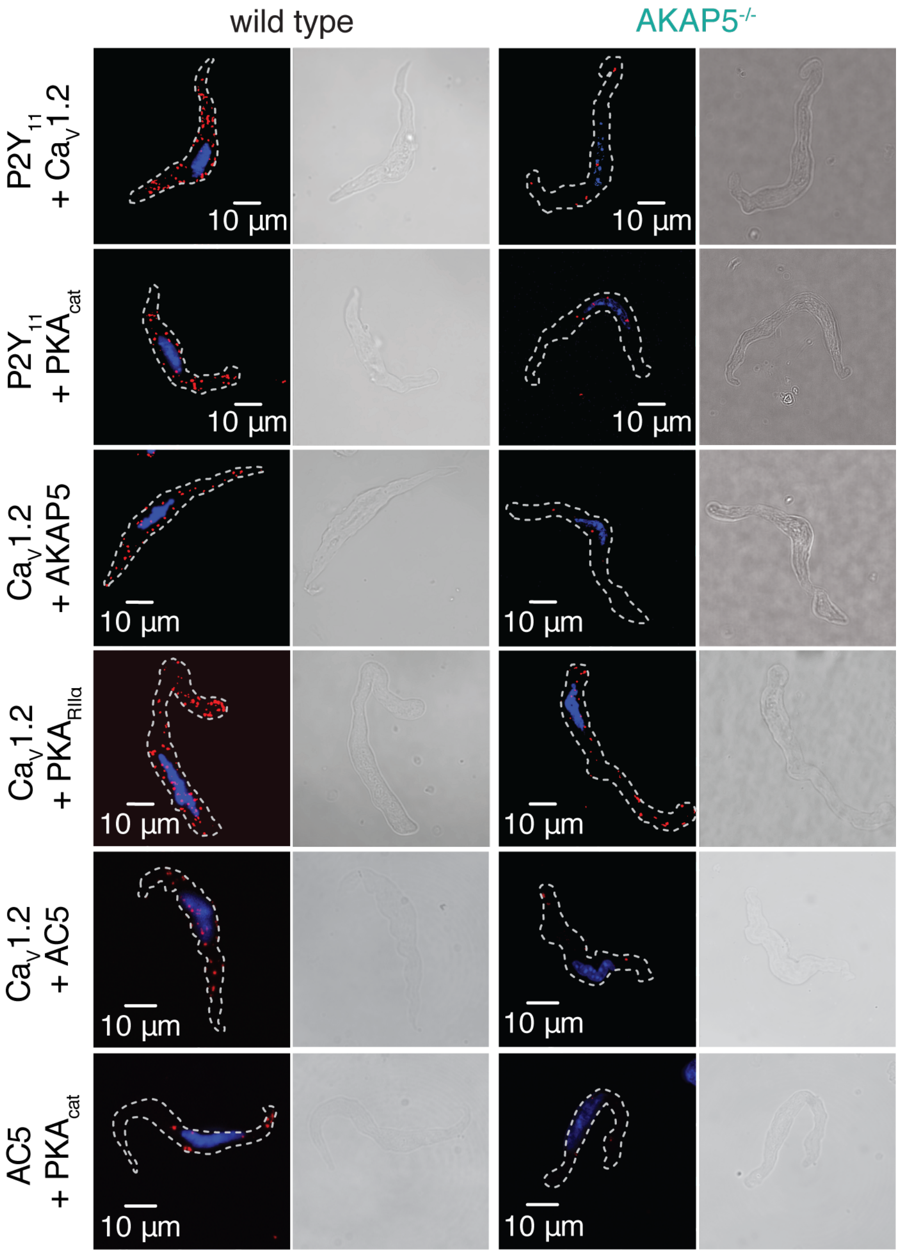

Supplementary Figure 8. PLA images for WT and AKAP5$^{-/-}$ arterial myocytes. Exemplary merged fluorescence proximity ligation assay (PLA; red)/nucleus (blue; left panels) and differential interference contrast (right panels) images of wild type (WT) and AKAP5$^{-/-}$ cerebral arterial myocytes co-labeled for P2Y$_{11}$ + Ca$_V$1.2, P2Y$_{11}$ + PKA$_{cat}$, Ca$_V$1.2 + AKAP5, Ca$_V$1.2 + PKA$_{RII\alpha}$, Ca$_V$1.2 + AC5 and AC5 + PKA$_{cat}$.

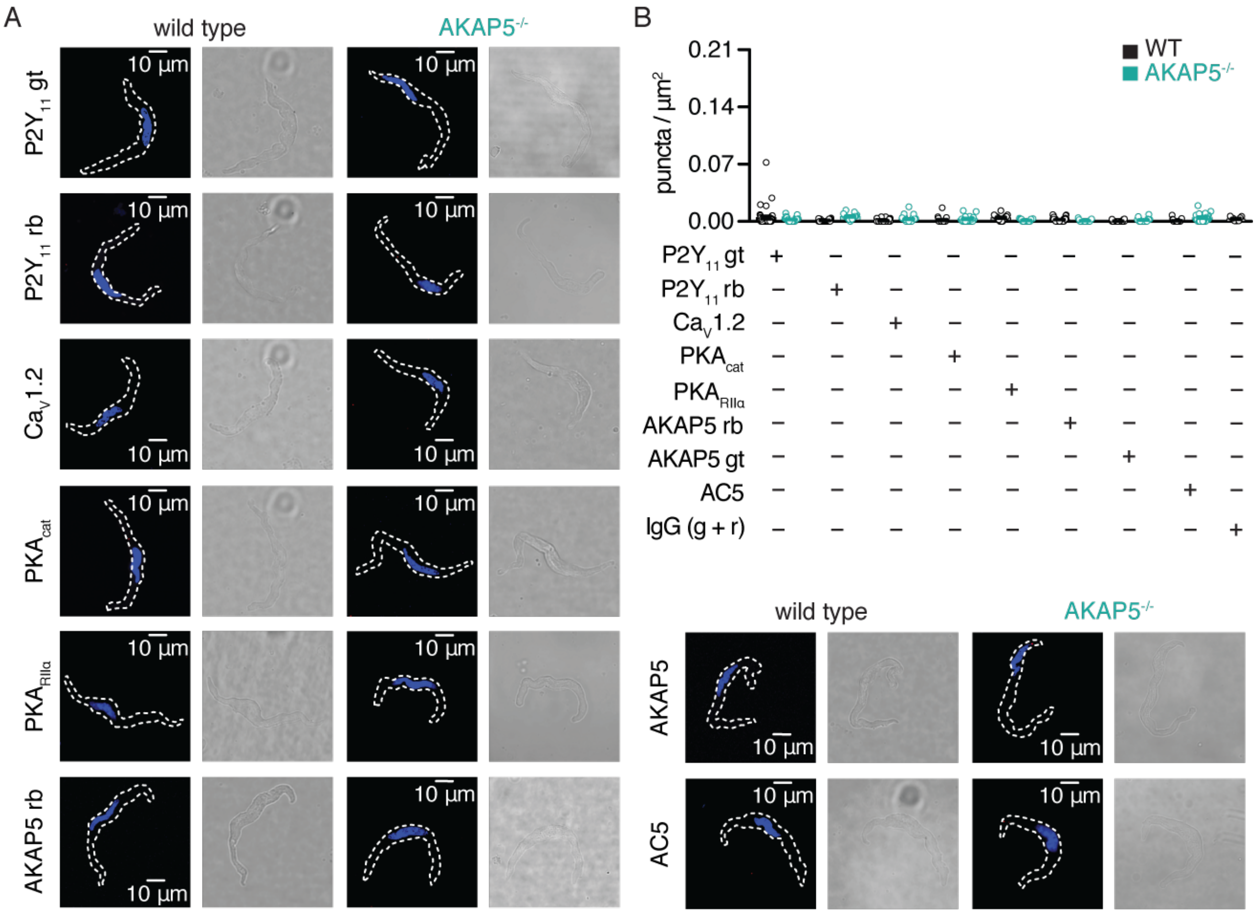

Supplementary Figure 9. PLA images and quantification of negative control experiments for WT and AKAP5$^{-/-}$ arterial myocytes. A) Representative merged fluorescence proximity ligation assay (PLA; red)/nucleus (blue; left panels) and differential interference contrast (right panels) images and **B)** quantification of PLA fluorescent puncta per cell area (puncta/μm^2) of wild type (WT) and AKAP5$^{-/-}$ arterial myocytes labeled with only one 1° antibody for P2Y$_{11}$ goat (gt; n = 43 cells/3 WT mice; n = 37 cells/3 AKAP5$^{-/-}$ mice), P2Y$_{11}$ rabbit (rb; n = 20 cells/3 WT mice; n = 17 cells/3 AKAP5$^{-/-}$ mice), Ca$_V$1.2 (n = 43 cells/3 WT mice; n = 34 cells/3 AKAP5$^{-/-}$ mice), PKA$_{cat}$ (n = 20 cells/3 WT mice; n = 18 cells/3 AKAP5$^{-/-}$ mice), PKA$_{RII\alpha}$ (n = 16 cells/3 WT mice; n = 15 cells/3 AKAP5$^{-/-}$ mice), AKAP5 rabbit (rb; n = 41 cells/3 WT mice; n = 15 cells/3 AKAP5$^{-/-}$ mice), AKAP5 goat (gt; n = 20 cells/3 WT mice; n = 18 cells/3 AKAP5$^{-/-}$ mice) and AC5 (n = 21 cells/3 WT mice; n = 26 cells/3 AKAP5$^{-/-}$ mice). For some experiments, WT arterial myocytes were labeled with primary goat and rabbit immunoglobulin G

(IgGs) followed by the PLUS and MINUS probes of the PLA assay (IgG (g + r) dataset; n = 9 cells/2 mice) as negative control experiments. Data represent mean ± SEM. Source data are provided as Source Data file.

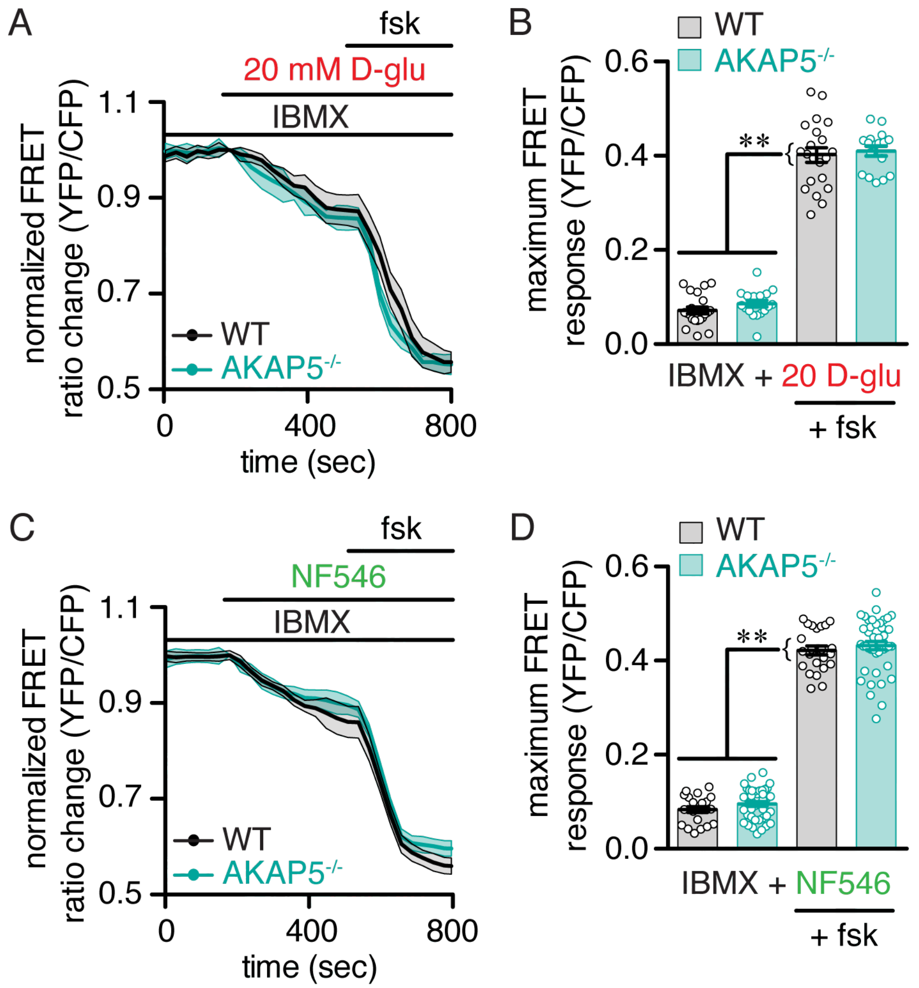

Supplementary Figure 10. PDE inhibition rescues glucose/NF546-induced cAMP synthesis in arterial myocytes. Time courses of the average ICUE3-PM traces (mean = solid line; SEM = shadow) and summary data of wild type (WT) and AKAP5$^{-/-}$ arterial myocytes pretreated with the broad phosphodiesterase (PDE) inhibitor 3-isobutyl-1-methylxanthine (IBMX) in response to **A, B)** 20 mM D-glucose (D-glu; n = 21 cells/3 WT mice and n = 17 arteries/3 AKAP5$^{-/-}$ mice) and **C, D)** NF546 (n = 23 cells/3 WT mice and n = 40 arteries/3 AKAP5$^{-/-}$ mice) and after application of forskolin. **P* < 0.05 with two-tailed Mann-Whitney test. The single asterisks highlight significant differences between datasets in the absence of forskolin. The double asterisks indicate a statistical difference within the same experimental group in the absence and presence forskolin. All significant *P* values are < 0.0001. Data represent mean ± SEM. Source data are provided as Source Data file.

Supplementary Table 1: Human nondiabetic patients undergoing surgical sleeve gastrectomy

age (years)	gender
41.5 ± 5.0	5 F / 3 M

Values are mean ± SEM. n = 8 samples obtained from 5 female and 3 male donors. Source data are provided as Source Data file.

Supplementary Table 2: Percent treatment-induced changes in tone for experiments in Figure 3A and Figure 3D.

	WT (3A)	AKAP5$^{-/-}$ (3A)	WT (3D)	AKAP5$^{-/-}$ (3D)	AC5$^{-/-}$ (3D)
**peak 60 mM K^+ constriction (%)**	67.4 ± 3.0	58.4 ± 8.0	62.2 ± 3.7	66.0 ± 2.9	69.9 ± 3.5
**arterial tone (%) in 10 mM D-glu**	16.1 ± 2.0	17.0 ± 1.7	14.6 ± 1.5	21.8 ± 3.4	15.9 ± 2.5
**arterial tone (%) in 20 mM D-glu (3A) or NF546 (3D)**	26.1 ± 2.7*	19.5 ± 1.3	24.3 ± 3.1*	21.7 ± 3.3	15.6 ± 2.6
**Δ arterial tone (%)**	10.0 ± 1.0*	2.4 ± 1.1	9.8 ± 2.2*	-0.1 ± 0.5	-0.2 ± 0.2

Values are mean ± SEM. For Figure 3A, n = 9 arteries from 5 WT mice and 7 arteries from 6 AKAP5$^{-/-}$ mice. For Figure 3D, n = 6 arteries from 4 WT mice, 8 arteries from 6 AKAP5$^{-/-}$ mice and 6 arteries from 4 AC5$^{-/-}$ mice. *$P < 0.05$ with two-tailed Wilcoxon test for data associated with Figure 3A and Kruskal-Wallis One-way ANOVA with Dunn's multiple comparisons for data associated with Figure 3D. $P = 0.0039$ for WT 10 mM D-glu-20 mM D-glu comparison in Figure 3Aii, $P = 0.0012$ for WT-AKAP5$^{-/-}$ comparison in Figure 3Aiii, $P = 0.0313$ for WT 10 mM D-glu-NF546 comparison in Figure 3Dii, and $P = 0.0036$ for WT-AKAP5$^{-/-}$ and $P = 0.0040$ for WT-AC5$^{-/-}$ for comparison in Figure 3Diii. Source data are provided as Source Data file.

Supplementary Table 3: Percent treatment-induced changes in Figure 4.

Percent treatment-induced changes in tone in Figure 4A and 4C		
	**WT**	**$AKAP5^{-/-}$**
**arterial tone (%) in 10 mM D-glu**	24.5 ± 2.8	22.5 ± 2.7
**arterial tone (%) in 10 mM D-glu + 10 mM mannitol**	24.4 ± 3.1	23.5 ± 3.1
**arterial tone (%) in 20 mM D-glu**	45.1 ± 2.6*	26.4 ± 2.8
**arterial tone (%) in 20 mM D-glu + NF546**	40.7 ± 1.8*	22.9 ± 2.2
**arterial tone (%) in 10 mM D-glu**	23.1 ± 2.6	-
**arterial tone (%) in NF340 + 10 mM D-glu + 10 mM mannitol**	24.1 ± 3.0	-
**arterial tone (%) in NF340 + 20 mM D-glu**	21.8 ± 2.1	-
**arterial tone (%) in NF340 + 20 mM D-glu + NF546**	24.6 ± 2.3	-
**Percent treatment-induced changes in blood flow in Figure 4B**		
**Δ blood flow in 10 mM D-glu + 10 mM mannitol**	0.91 ± 0.01	1.01 ± 0.01
**Δ in 20 mM D-glu**	0.80 ± 0.02*	0.97 ± 0.01
**Δ in 20 mM D-glu + NF546**	0.77 ± 0.03*	1.00 ± 0.01

Values are mean ± SEM. For Figure 4A, n = 18 arteries from 3 WT mice and n = 21 arteries from 3 $AKAP5^{-/-}$ mice. For Figure 4B, n = 18 arteries from 5 WT mice and 12 arteries from 3 $AKAP5^{-/-}$ mice. For Figure 4C, n = 23 arteries from 3 WT mice. *$P < 0.05$ with Friedman One-way ANOVA with Dunn's multiple comparisons. $P < 0.0001$ for WT 10 mM D-glu-20 mM D-glu and $P = 0.0004$ for WT 10 mM D-glu-20 mM D-glu + NF546 for comparisons in Figure 4A, and $P = 0.0007$ for WT mannitol-20 mM D-glu and $P < 0.0001$ for WT mannitol-20 mM D-glu + NF546 for comparisons in Figure 4B. Source data are provided as Source Data file.

Supplementary Table 4: Percent treatment-induced changes in tone for experiments in Figure 7B.

	WT	$AKAP5^{-/-}$
**peak 60 mM K^+ constriction (%)**	66.7 ± 3.0	59.9 ± 3.7
**arterial tone (%) in IBMX + 10 mM D-glu**	15.7 ± 3.1	15.3 ± 2.1
**arterial tone (%) in IBMX + 20 mM D-glu**	26.5 ± 2.9*	15.7 ± 2.3
**arterial tone (%) in IBMX + 20 mM D-glu + NF546**	26.5 ± 2.9*	15.6 ± 2.4

Values are mean ± SEM. For Figure 7B, n = 6 arteries from 4 WT mice and 6 arteries from 3 $AKAP5^{-/-}$ mice. *$P < 0.05$ with two-tailed Mann-Whitney test for 60 mM K^+ response and Friedman One-way ANOVA with Dunn's multiple comparisons for multiple comparisons. $P = 0.0281$ for WT 10 mM D-glu + IBMX-20 mM D-glu + IBMX and $P = 0.0281$ for WT 10 mM D-glu + IBMX-20 mM D-glu + NF546 + IBMX for comparisons in Figure 7B. Source data are provided as Source Data file.

Supplementary Table 5: Key reagents/resources used in the study.

Reagent type (species) or resource	Designation	Source or reference	Identifiers	Additional Information
strain (*Mus musculus*), C57BL/6J	wild-type	Jackson Laboratories	stock # 000664	
strain (*Mus musculus*)	AKAP5$^{-/-}$	[1,2]		Backcrossed for 10 generations into the C57BL/6J background
strain (*Mus musculus*)	AC5$^{-/-}$	[3]		Backcrossed for 10 generations into the C57BL/6J background
antibody	FP1 (Ca_V1.2; custom rabbit)	[4]		1:100 dilution. Used for mouse datasets. Validated in Davare et al, JBC doi: 10.1074/jbc.M005462200 and Buonarati et al F1000Res doi: 10.12688/f1000research.11808.2
antibody	anti-Ca_V1.2 (mouse monoclonal)	Neuromab	clone N263/31	1:200 dilution. Used for human datasets. For validation see http://neuromab.ucdavis.edu/datasheet/N263_31.pdf
antibody	anti-AC5 (goat polyclonal)	Santa Cruz Biotechnology	sc74301 RRID: AB_2289217	1:50-1:1000 dilutions. Validated in Syed et al, JCI doi: 10.1172/JCI124705
antibody	anti-$P2Y_{11}$ (rabbit polyclonal)	Abcam	ab180739	1:100-1:200 dilutions. Validated in Prada et al. eLife doi: 10.7554/eLife.42214
antibody	anti-$P2Y_{11}$ (goat polyclonal)	Santa Cruz Biotechnology	sc-69588; clone C-18	1:100-1:200 dilutions. Prada et al. eLife doi:

			RRID: AB_21559	10.7554/eLife.42214
antibody	anti-AKAP79 (rabbit polyclonal)	Millipore	ABS102	1:200 dilution. For validation see https://www.emdmillipore.com/US/en/product/Anti-AKAP-79-Antibody,MM_NF-ABS102?ReferrerURL=https%3A%2F%2Fwww.google.com%2F
antibody	anti-AKAP150 (rabbit polyclonal)	Millipore	07-210	1:200 dilution. Validated in Nystoriak et al, Science Signaling doi: 10.1126/scisignal.aaf9647
antibody	anti-AKAP150 (goat polyclonal)	Santa Cruz Biotechnology	clone C-20	1:200 dilution. For validation see https://www.scbt.com/p/akap-150-antibody-c-20?productCanUrl=akap-150-antibody-c-20&_requestid=2603832,; additional information in https://datasheets.scbt.com/sc-377055.pdf
antibody	anti-$PKA_{RII\alpha}$ (mouse monoclonal)	BD Transduction Laboratories	612242	1:50 dilution. Validated in Prada et al. eLife doi: 10.7554/eLife.42214
antibody	anti-PKA_{cat} α, β, γ (rabbit polyclonal)	Santa Cruz Biotechnology	sc-28892; clone H-95	1:200 dilution. For validation see http://datasheets.scbt.com/sc-28892.pdf
antibody	goat anti-rabbit IgG (H+L)-	Bio-Rad	170-6515 RRID: AB_11125142	1:10000 dilution. For validation see https://www.bio-

	horseradish peroxidase conjugate			rad.com/en-us/sku/1706515-goat-anti-rabbit-igg-h-l-hrp-conjugate?ID=1706515#
antibody	IRDye 800CW goat anti-mouse/rabbit	Abcam	ab216772/ ab216773	1:5000. For validation see https://www.abcam.com/goat-mouse-igg-hl-irdyereg-800cw-preadsorbed-ab216772.html and https://www.abcam.com/goat-rabbit-igg-hl-irdyereg-800cw-preadsorbed-ab216773.html
antibody	Alexa Fluor 430-conjugated goat anti-rabbit	Molecular Probes	A11064 RRID: AB_2534111	5 mg/mL dilution. For validation see https://www.thermofisher.com/antibody/product/Goat-anti-Rabbit-IgG-H-L-Cross-Adsorbed-Secondary-Antibody-Polyclonal/A-11064
antibody	Alexa Fluor 568-conjugated donkey anti-mouse	Molecular Probes	A10037 RRID: AB_2534013	5 µg/mL dilution. For validation see Prada et al. eLife doi: 10.7554/eLife.42214; Nystoriak et al, Science Signaling doi: 10.1126/scisignal.aaf9647; Syed et al, JCI doi: 10.1172/JCI124705; https://www.thermofisher.com/antibody/product/Donkey-anti-Mouse-IgG-H-L-Highly-

				Cross-Adsorbed-Secondary-Antibody-Polyclonal/A10037
antibody	Alexa Fluor 647-conjugated donkey anti-goat	Molecular Probes	A21447 RRID: 141844	5 µg/mL dilution. For validation see Prada et al. eLife doi: 10.7554/eLife.42214; Nystoriak et al, Science Signaling doi: 10.1126/scisignal.aaf9647; Syed et al, JCI doi: 10.1172/JCI124705; https://www.thermofisher.com/antibody/product/Donkey-anti-Goat-IgG-H-L-Cross-Adsorbed-Secondary-Antibody-Polyclonal/A-21447
antibody	normal mouse IgG	Millipore	NI03	10 µg/mL dilution. For validation see https://www.emdmillipore.com/US/en/product/Normal-Mouse-IgG,EMD_BIO-NI03
antibody	normal rabbit IgG	Cell Signaling	2729S	10 µg/mL dilution. For validation see https://www.cellsignal.com/products/primary-antibodies/normal-rabbit-igg/2729
antibody	normal goat IgG	Millipore	NI02	10 µg/mL dilution. For validation see https://www.emdmillipore.com/US/en/product/Normal-Goat-

				IgG,EMD_BIO-NI02
antibody	CD31 - biotin	BD Bioscience	558737 RRID: AB_397096	5 mg/mL dilution. For validation see https://www.bdbiosciences.com/eu/applications/research/stem-cell-research/cancer-research/mouse/biotin-rat-anti-mouse-cd31-390/p/558737
antibody	CD45 - biotin	BD Bioscience	553077 RRID: AB_394607	2 µg/mL dilution. For validation see https://www.bdbiosciences.com/eu/applications/research/stem-cell-research/cancer-research/mouse/biotin-rat-anti-mouse-cd45-30-f11/p/553077
antibody	lineage antibody cocktail	BD Bioscience	559971 RRID: AB_10053179	2 µg/mL dilution. For validation see https://www.bdbiosciences.com/eu/applications/research/stem-cell-research/stem-cell-kits-and-cocktails/mouse/biotin-mouse-lineage-panel/p/559971
FRET biosensor	ICUE3-PM/ ICUE3-NLS	[5,6]		viruses/DNA constructs
chemical compound, drug	IBMX	Sigma	I5879	
chemical compound, drug	mannitol	Fisher Scientific	BP686	
chemical compound, drug	NF340	Santa Cruz Biotechnology	sc-361274	
chemical compound, drug	NF546	Tocris	3892	
chemical compound, drug	nifedipine	Sigma-Aldrich	N7634	
chemical compound, drug	forskolin	Sigma-Aldrich	F6886	

chemical compound, drug	amphotericin B	Sigma-Aldrich	A4888	
chemical compound, drug	Duolink	Sigma	[7]	
software, algorithm	GraphPad Prism		GraphPad Prism, RRID: SCR_002798	v6
software, algorithm	Origin	OriginLab		v7.0
software, algorithm	ImageJ		Fiji, RRID: SCR_002285	v1.51
software, algorithm	pCLAMP10	Molecular Devices		v10.3; electrophysiology
software, algorithm	IonOptix	IonOptix		v6.6; arterial diameter recordings
software, algorithm	Metaflor	Molecular Devices		v7.7; FRET
software, algorithm	StCamSware	Sentech America Incorporated		v3.10.0.3086
hardware	Laser Speckle Contrast Imager	Moor Instruments	FLPI-2	
software, algorithm	Moor FLPI Review	Moor Instruments	FLPI	v5.0
software, algorithm	LASAF	Leica		for GSD imaging
software, algorithm	Zen	Zeiss		v2.3 SP1; for Airyscan imaging
software, algorithm	Fluoview	Olympus		v1.4
software, algorithm	CellQuest	BD Biosciences		v5.2.1
software, algorithm	FlowJo	Treestar Inc		v9.9.6

Supplementary References

1. Navedo, M.F., *et al.* AKAP150 is required for stuttering persistent Ca2+ sparklets and angiotensin II-induced hypertension. *Circ Res* **102**, e1-e11 (2008).
2. Nichols, C.B., *et al.* Sympathetic stimulation of adult cardiomyocytes requires association of AKAP5 with a subpopulation of L-type calcium channels. *Circ Res* **107**, 747-756 (2010).
3. Timofeyev, V., *et al.* Adenylyl cyclase subtype-specific compartmentalization: differential regulation of L-type Ca2+ current in ventricular myocytes. *Circ Res* **112**, 1567-1576 (2013).
4. Davare, M.A., Horne, M.C. & Hell, J.W. Protein phosphatase 2A is associated with class C L-type calcium channels (Cav1.2) and antagonizes channel phosphorylation by cAMP-dependent protein kinase. *J Biol Chem* **275**, 39710-39717 (2000).
5. Allen, M.D., *et al.* Reading dynamic kinase activity in living cells for high-throughput screening. *ACS chemical biology* **1**, 371-376 (2006).
6. Liu, S., Zhang, J. & Xiang, Y.K. FRET-based direct detection of dynamic protein kinase A activity on the sarcoplasmic reticulum in cardiomyocytes. *Biochemical and biophysical research communications* **404**, 581-586 (2011).
7. Fredriksson, S., *et al.* Protein detection using proximity-dependent DNA ligation assays. *Nature biotechnology* **20**, 473-477 (2002).
