# Supplementary material for: AKAP5 complex facilitates purinergic modulation of vascular L-type Ca2+ channel CaV1.2
Source: Nat Commun. 2020 Oct 20;11:5303. doi: 10.1038/s41467-020-18947-y (PMC7575592; doi:10.1038/s41467-020-18947-y)
Supplement: Supplementary file 3 — Reporting Summary [file 41467_2020_18947_MOESM3_ESM.pdf]

nature research

Corresponding author(s): Madeline Nieves-Cintron
Manuel F. Navedo

Last updated by author(s): Aug 31, 2020

Reporting Summary

Nature Research wishes to improve the reproducibility of the work that we publish. This form provides structure for consistency and transparency in reporting. For further information on Nature Research policies, see Authors & Referees and the Editorial Policy Checklist.

Statistics

For all statistical analyses, confirm that the following items are present in the figure legend, table legend, main text, or Methods section.

n/a	Confirmed	
☐	☒	The exact sample size (*n*) for each experimental group/condition, given as a discrete number and unit of measurement
☐	☒	A statement on whether measurements were taken from distinct samples or whether the same sample was measured repeatedly
☐	☒	The statistical test(s) used AND whether they are one- or two-sided<br>*Only common tests should be described solely by name; describe more complex techniques in the Methods section.*
☐	☒	A description of all covariates tested
☐	☒	A description of any assumptions or corrections, such as tests of normality and adjustment for multiple comparisons
☐	☒	A full description of the statistical parameters including central tendency (e.g. means) or other basic estimates (e.g. regression coefficient) AND variation (e.g. standard deviation) or associated estimates of uncertainty (e.g. confidence intervals)
☐	☒	For null hypothesis testing, the test statistic (e.g. *F*, *t*, *r*) with confidence intervals, effect sizes, degrees of freedom and *P* value noted<br>*Give P values as exact values whenever suitable.*
☒	☐	For Bayesian analysis, information on the choice of priors and Markov chain Monte Carlo settings
☒	☐	For hierarchical and complex designs, identification of the appropriate level for tests and full reporting of outcomes
☒	☐	Estimates of effect sizes (e.g. Cohen's *d*, Pearson's *r*), indicating how they were calculated

Our web collection on statistics for biologists contains articles on many of the points above.

Software and code

Policy information about availability of computer code

Data collection	Metaflor v7.7, Clampex v10.3, IonOptix v6.6, Moor FLPI-2, StCamSWare v3.10.0.3086, Olympus Fluoview v1.4, Leica LASAF, Zeiss Zen v2.3 PS1 with an Airyscan detector module, CellQuest v5.2.1.
Data analysis	Metaflor v7.7, IonOptix v6.6, Moor FLPI v5.0, StCamSWare v3.10.0.3086, Clampfit v10.3, ImageJ v1.51, GraphPad Prism v6.0, Origin v7.0, Zeiss Zen v2.3 SP1, Leica LASAF, FlowJo v9.9.6

For manuscripts utilizing custom algorithms or software that are central to the research but not yet described in published literature, software must be made available to editors/reviewers. We strongly encourage code deposition in a community repository (e.g. GitHub). See the Nature Research guidelines for submitting code & software for further information.

Data

Policy information about availability of data

All manuscripts must include a data availability statement. This statement should provide the following information, where applicable:

- Accession codes, unique identifiers, or web links for publicly available datasets
- A list of figures that have associated raw data
- A description of any restrictions on data availability

All data generated or analyzed in this study are included in the main manuscript and/or supplementary figures. Source data are provided as Source Data file. All other raw data is available upon request.

Field-specific reporting

Please select the one below that is the best fit for your research. If you are not sure, read the appropriate sections before making your selection.

☒ Life sciences ☐ Behavioural & social sciences ☐ Ecological, evolutionary & environmental sciences

For a reference copy of the document with all sections, see nature.com/documents/nr-reporting-summary-flat.pdf

Life sciences study design

All studies must disclose on these points even when the disclosure is negative.

Sample size	We did not perform an a priori sample size calculation. However, all experimental series used technical replicates (not biological replicates) as they examined acute effects. The number of such replicates is based on previous published observations to reached statistical differences between similar datasets (as in Navedo, M.F., et al. Circ Res 102, e1-e11, Navedo, M.F., et al. Am J Physiol Cell Physiol 298, C211-220. PMC2822492, Nieves-Cintron, M., et al. JBC 290, 7918-7929. PMC4367290, Nieves-Cintron, M., et al. Scientific Reports 7, 14058. PMC5656614, Nystoriak, M.A., et al. Circ Res 114, 607-615. PMC3954117, Nystoriak, M.A., et al. Science Signaling 10, 7. PMC5297430, Prada, M.P., et al. eLife 8: e42214. PMC6397001). To account for variability in sample preparation, animals, ambience conditions, etc, datasets were produced from cells obtained from at least 2 different mice/human samples.
Data exclusions	No data was excluded.
Replication	To account for variability in sample preparation, animals, ambience conditions, etc, datasets are produced from cells from at least 2 different mice/human samples. All attempts at replication were successful.
Randomization	Randomization was not relevant for the current study. Treatments were acutely applied in cells/tissues that were randomly selected.
Blinding	Blinding was not possible for all experiments as they were performed before and after a specific acute treatment. Personnel performing PLA, in vivo arterial diameter measurements and blood flow analysis was blinded to the experimental condition.

Reporting for specific materials, systems and methods

We require information from authors about some types of materials, experimental systems and methods used in many studies. Here, indicate whether each material, system or method listed is relevant to your study. If you are not sure if a list item applies to your research, read the appropriate section before selecting a response.

Materials & experimental systems

n/a	Involved in the study
☐	☒ Antibodies
☒	☐ Eukaryotic cell lines
☒	☐ Palaeontology
☐	☒ Animals and other organisms
☒	☐ Human research participants
☒	☐ Clinical data

Methods

n/a	Involved in the study
☒	☐ ChIP-seq
☐	☒ Flow cytometry
☒	☐ MRI-based neuroimaging

Antibodies

Antibodies used

Custom rabbit polyclonal anti-Cav1.2 (Davare et al. JBC 275, 39710-39717; 1:100)
Mouse monoclonal anti-Cav1.2 (Neuromab; clone N263/31; 1:200)
Goat polyclonal anti-AC5 (Santa Cruz Biotechnology; sc74301; 1:50-1:1000)
Rabbit polyclonal anti-P2Y11 (Abcam ab180739 1:100-1:200)
Goat polyclonal anti-P2Y11 (Santa Cruz Biotechnology; sc-69588 - clone C-18; 1:100-1:200)
Rabbit polyclonal anti-AKAP79 (Millipore ABS102; 1:200)
Rabbit polyclonal anti-AKAP150 (Millipore 07-210; 1:200)
Goat polyclonal anti-AKAP150 (Santa Cruz Biotechnology; clone C-20; 1:200)
Mouse monoclonal anti-PKARIIα (BD Transduction Laboratories; 612242; 1:50)
Rabbit polyclonal anti-PKAcat α, β, γ (Santa Cruz Biotechnology; sc-28892; clone H-95 1:200)
Goat anti-rabbit IgG (H+L)-horseradish peroxidase conjugate (Bio-Rad; 170-6515; 1:10000)
Goat anti-mouse/rabbit IRDye 800CW (Abcam; ab216772/ab216773; 1:5000)
Goat anti-rabbit Alexa Fluor 430 (Molecular Probes; A11064; 5 mg/mL)
Donkey anti-mouse Alexa Fluor 568 (Molecular Probes; A10037; 5 µg/mL)
Donkey anti-goat Alexa Fluor 647 (Molecular Probes; A21447; 5 µg/mL)
normal mouse IgG (Millipore; NI03; 10 µg/mL)
normal rabbit IgG (Cell Signaling; 2729S; 10 µg/mL)
normal goat IgG (Millipore; NI02; 10 µg/mL)

	CD31 - biotin (BD Bioscience; 558737; 5 mg/mL)<br>CD45 - biotin (BD Bioscience; 553077; 2 µg/mL)<br>lineage antibody cocktail (BD Bioscience; 559971; 2 µg/mL)
Validation	All antibodies are commercially available or upon request by source, and the applications have been tested by the manufacturer and by us in different studies<br><br>The FP1 antibody was generated and validated by Dr. Johannes W. Hell via multiple methods including KO, different epitopes, over-expression, etc (Davare et al, JBC doi: 10.1074/jbc.M005462200 and Buonarati et al F1000Res doi: 10.12688/f1000research.11808.2)<br><br>Mouse monoclonal anti-Cav1.2 (http://neuromab.ucdavis.edu/datasheet/N263_31.pdf)<br><br>Goat polyclonal anti-AC5 was validated using AC5 KO tissue (Syed et al, JCI doi: 10.1172/JCI124705)<br><br>Anti-P2Y11 were validated using KO and over-expression in tsA-201 cells (Prada et al. eLife doi: 10.7554/eLife.42214)<br><br>Rabbit polyclonal anti-AKAP79 (https://www.emdmillipore.com/US/en/product/Anti-AKAP-79-Antibody,MM_NF-ABS102?ReferrerURL=https%3A%2F%2Fwww.google.com%2F)<br><br>Rabbit polyclonal anti-AKAP150 was validated with AKAP5 KO tissue (Nystoriak et al, Science Signaling doi: 10.1126/scisignal.aaf9647)<br><br>Goat polyclonal anti-AKAP150 (https://www.scbt.com/p/akap-150-antibody-c-20?productCanUrl=akap-150-antibody-c-20&_requestid=2603832,; additional information in https://datasheets.scbt.com/sc-377055.pdf)<br><br>Mouse monoclonal anti-PKARIIα was validated by using pre-absorbed samples (Prada et al. eLife doi: 10.7554/eLife.42214)<br><br>Rabbit polyclonal anti-PKAcat α, β, γ (http://datasheets.scbt.com/sc-28892.pdf)<br><br>Goat anti-rabbit IgG (H+L)-horseradish peroxidase conjugate (https://www.bio-rad.com/en-us/sku/1706515-goat-anti-rabbit-igg-h-l-hrp-conjugate?ID=1706515#)<br><br>Goat anti-mouse/rabbit IRDye 800CW (https://www.abcam.com/goat-mouse-igg-hl-irdyereg-800cw-preadsorbed-ab216772.html and https://www.abcam.com/goat-rabbit-igg-hl-irdyereg-800cw-preadsorbed-ab216773.html)<br><br>Goat anti-rabbit Alexa Fluor 430 (https://www.thermofisher.com/antibody/product/Goat-anti-Rabbit-IgG-H-L-Cross-Adsorbed-Secondary-Antibody-Polyclonal/A-11064)<br><br>Donkey anti-mouse Alexa Fluor 568 (Prada et al. eLife doi: 10.7554/eLife.42214; Nystoriak et al, Science Signaling doi: 10.1126/scisignal.aaf9647; Syed et al, JCI doi: 10.1172/JCI124705; https://www.thermofisher.com/antibody/product/Donkey-anti-Mouse-IgG-H-L-Highly-Cross-Adsorbed-Secondary-Antibody-Polyclonal/A10037)<br><br>Donkey anti-goat Alexa Fluor 647 (Prada et al. eLife doi: 10.7554/eLife.42214; Nystoriak et al, Science Signaling doi: 10.1126/scisignal.aaf9647; Syed et al, JCI doi: 10.1172/JCI124705; https://www.thermofisher.com/antibody/product/Donkey-anti-Goat-IgG-H-L-Cross-Adsorbed-Secondary-Antibody-Polyclonal/A-21447)<br><br>normal mouse IgG (https://www.emdmillipore.com/US/en/product/Normal-Mouse-IgG,EMD_BIO-NI03)<br><br>normal rabbit IgG (https://www.cellsignal.com/products/primary-antibodies/normal-rabbit-igg/2729)<br><br>normal goat IgG (https://www.emdmillipore.com/US/en/product/Normal-Goat-IgG,EMD_BIO-NI02)<br><br>CD31 - biotin (https://www.bdbiosciences.com/eu/applications/research/stem-cell-research/cancer-research/mouse/biotin-rat-anti-mouse-cd31-390/p/558737)<br><br>CD45 - biotin (https://www.bdbiosciences.com/eu/applications/research/stem-cell-research/cancer-research/mouse/biotin-rat-anti-mouse-cd45-30-f11/p/553077)<br><br>lineage antibody cocktail (https://www.bdbiosciences.com/eu/applications/research/stem-cell-research/stem-cell-kits-and-cocktails/mouse/biotin-mouse-lineage-panel/p/559971)

Animals and other organisms

Policy information about studies involving animals; ARRIVE guidelines recommended for reporting animal research

Laboratory animals	Age-matched (5-8 weeks) male and female C57BL/6J (WT), male AKAP5-/- and male AC5-/- mice were used in this study.
Wild animals	The study did not involve wild animals.
Field-collected samples	The study did not involve any field-collected samples.

Ethics oversight	Animals studies were approved and overseen by the University of California Davis Animal Care and Use Committee (protocols #: 20321 and 20234).

Note that full information on the approval of the study protocol must also be provided in the manuscript.

Flow Cytometry

Plots

Confirm that:

☒ The axis labels state the marker and fluorochrome used (e.g. CD4-FITC).

☒ The axis scales are clearly visible. Include numbers along axes only for bottom left plot of group (a 'group' is an analysis of identical markers).

☒ All plots are contour plots with outliers or pseudocolor plots.

☒ A numerical value for number of cells or percentage (with statistics) is provided.

Methodology

Sample preparation	Single cells were filtered through 200 µm cell strainer, fixed in 0.4% paraformaldehyde and treated with antibodies.
Instrument	Data was collected using FACScan cytometer (BD Biosciences, San Jose, CA) upgraded to a dual laser system with the addition of a blue laser (15mW at 488nm) and a red laser (25mW at 637nm Cytek Development, Inc, Fremont, CA).
Software	Data was acquired and analyzed using CellQuest (ver5.2.1 BD Bioscience) and FlowJo software (ver9.9.6 Treestar Inc., San Carlos) respectively.
Cell population abundance	The purity of the smooth muscle cells was determined to be 89.2±0.4% of the cultured cells. Smooth muscle cells were identified as non-fibroblasts cells (Thy1.2 negative), α-smooth muscle actin positive and CD31-/CD45-/Lineage marker negative.
Gating strategy	Nucleated cells are selected from the mixed population based on the incorporation of 7-AAD. Fibroblasts were gated using Thy1.2 antibody. From the Thy1.2 negative fibroblasts, smooth muscle cells were identified as α-smooth muscle actin positive and CD31-, CD45- and lineage negative cells. Secondary antibodies were used to control for background fluorescence and to identify the positive and negative populations. The boundaries for the positive population was set at <1% using background fluorescence.

☒ Tick this box to confirm that a figure exemplifying the gating strategy is provided in the Supplementary Information.
